# MET∆14 promotes a ligand-dependent, AKT-driven invasive growth

**DOI:** 10.26508/lsa.202201409

**Published:** 2022-05-30

**Authors:** Marina Cerqua, Orsola Botti, Maddalena Arigoni, Noemi Gioelli, Guido Serini, Raffaele Calogero, Carla Boccaccio, Paolo M Comoglio, Dogus M Altintas

**Affiliations:** 1 Istituto Fondazione di Oncologia Molecolare - La Fondazione Italiana per la Ricerca sul Cancro (IFOM - FIRC) Institute of Molecular Oncology, Milano, Italy; 2 Department of Molecular Biotechnology and Health Sciences, University of Torino, Torino, Italy; 3 Candiolo Cancer Institute-Fondazione del Piemonte per l’Oncologia, Istituto di Ricovero e Cura a Carattere Scientifico, Candiolo, Italy; 4 Department of Oncology, University of Torino School of Medicine, Turin, Italy; 5 Laboratory of Cancer Stem Cell Research, Candiolo Cancer Institute, Fondazione Piemontese per Oncologia - Istituti di Ricovero e Cura a Carattere Scientifico (FPO-IRCCS), Turin, Italy; 6 Department of Oncology, University of Turin Medical School, Turin, Italy

## Abstract

*MET* is an oncogene encoding the tyrosine kinase receptor for hepatocyte growth factor (HGF). Upon ligand binding, MET activates multiple signal transducers, including PI3K/AKT, STAT3, and MAPK. When mutated or amplified, *MET* becomes a “driver” for the onset and progression of cancer. The most frequent mutations in the *MET* gene affect the splicing sites of exon 14, leading to the deletion of the receptor’s juxtamembrane domain (MET∆14). It is currently believed that, as in gene amplification, MET∆14 kinase is constitutively active. Our analysis of MET in carcinoma cell lines showed that MET∆14 strictly depends on HGF for kinase activation. Compared with wt MET, ∆14 is sensitive to lower HGF concentrations, with more sustained kinase response. Using three different models, we have demonstrated that MET∆14 activation leads to robust phosphorylation of AKT, leading to a distinctive transcriptomic signature. Functional studies revealed that ∆14 activation is predominantly responsible for enhanced protection from apoptosis and cellular migration. Thus, the unique HGF-dependent ∆14 oncogenic activity suggests consideration of HGF in the tumour microenvironment to select patients for clinical trials.

## Abstract (precis)

The *MET* oncogene’s most frequent mutation is exon14 deletion. The resulting MET∆14 kinase is not constitutively active but requires HGF. The response to the ligand is stronger and long-lasting, protecting cancer cells from apoptosis and driving invasive growth.

## Introduction

*MET* is an oncogene encoding the tyrosine kinase receptor for hepatocyte growth factor (HGF) (Galimi et al, 1993). Upon ligand binding, MET recruits multiple signal transducers, including PI3K/AKT (i.e., migration), STAT3 (i.e., differentiation), and MAPK (i.e., proliferation) (Ponzetto et al, 1993, 1994; Boccaccio et al, 1998). Upon gene amplification and/or mutations *MET* is altered in multiple cancer types. Exacerbated MET activity confers a functional advantage to cancer cells, unleashing invasive growth (de Luca et al, 1999; Boccaccio & Comoglio, 2006). Surprisingly, the most common mutations observed in cancer patients are not activating mutations of the kinase domain but mutations affecting the splice sites of exon 14. The latter result in exon 14 “skipping” from mRNA with complete deletion of the protein’s juxtamembrane domain (MET∆14) (Reungwetwattana & Ou, 2015). It was proposed that the juxtamembrane domain promotes MET degradation, acting as a negative regulator for MET activation (Lee & Yamada, 1994; Villa-Moruzzi et al, 1998; Peschard et al, 2001; Lee et al, 2006; Tan et al, 2010). Therefore, as for *MET* gene amplification, it was suggested that exon 14 “skipping” induces an uncontrolled, autonomous activation of MET and invasive growth. However, drugs targeting MET in patients in clinical trials yielded positive but puzzling results (Paik et al, 2015, 2020; Engstrom et al, 2017; Klempner et al, 2017; Hu et al, 2018; Landi et al, 2019; Moro-Sibilot et al, 2019; Drilon et al, 2020; Wolf et al, 2020). Specifically, only half of the patients harbouring MET∆14 benefited from MET-targeted therapies, suggesting that there are critical aspects of MET∆14 that remained unaccounted for Salgia et al (2020) and Fujino et al (2021).

Here we show that deletion of exon 14 does not result in constitutive activation of the kinase as *MET* amplification/activating mutations do. Upon HGF binding, the pathways triggered by MET∆14 and the transcriptional response are different from those elicited by ligand stimulation or amplification of wt MET. Hence, MET∆14 acts as an HGF-dependent gain-of-function mutation driving a robust and selective AKT activation, rendering cancer cells more prone to survival and migration. These observations may have important mechanistic implications for cancer with MET∆14 mutation.

## Results and Discussion

**Figure 1.**
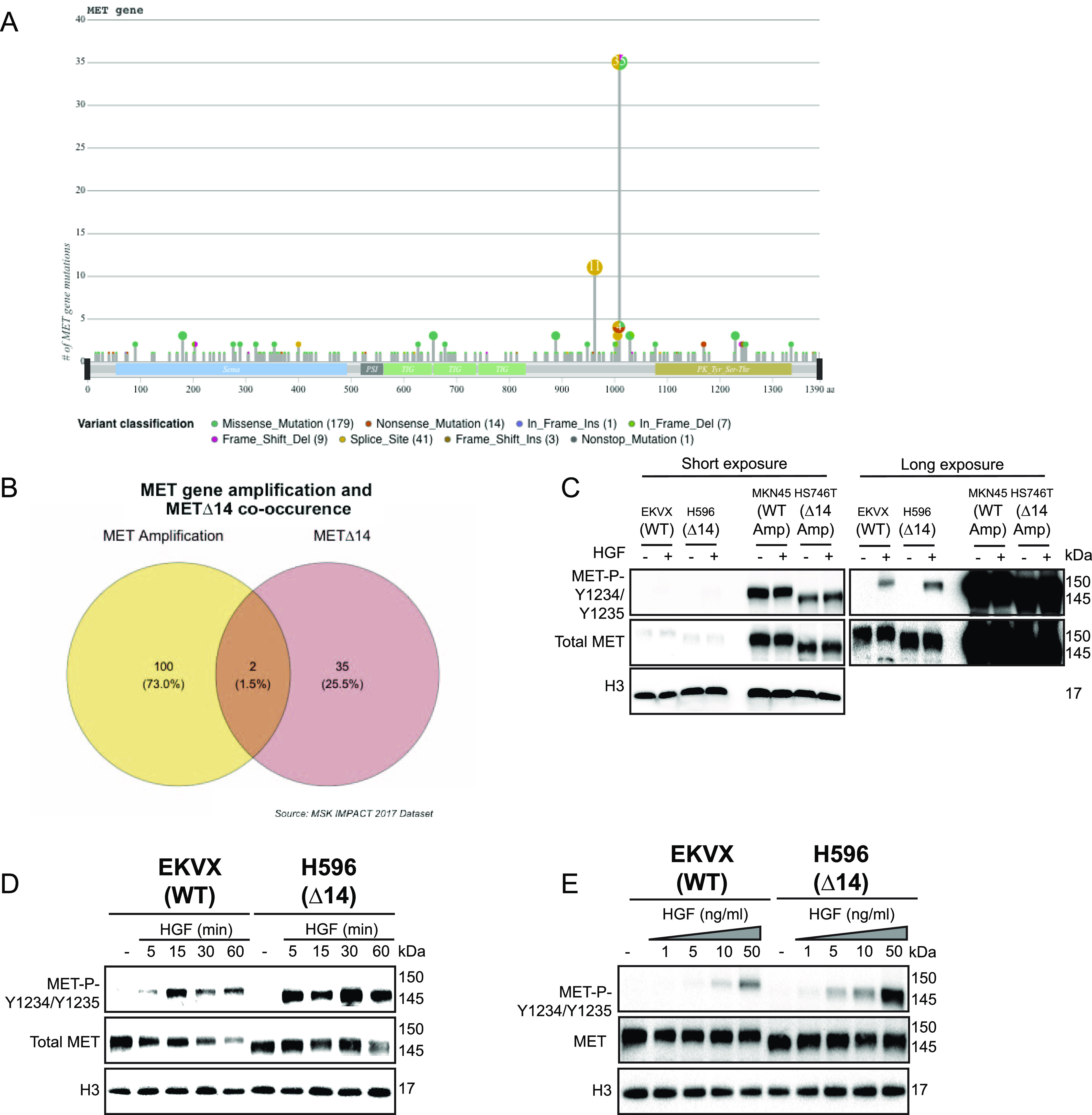
MET∆14 activation requires hepatocyte growth factor (HGF). **(A)** Number of mutations in the *MET* gene based on the MSK-IMPACT dataset (N > 10,000 patients) (Zehir et al, 2017). **(B)** Venn diagram showing cooccurrence of *MET* amplification and MET∆14 in patients published in MSK-IMPACT dataset (Zehir et al, 2017). **(C)** Immunoblot experiments using protein extracts from cell lines harbouring indicated MET alterations, cultivated in serum-free medium ± HGF. **(D)** Kinetics of activation of wt MET and MET∆14 after the stated time of HGF treatment, assessed by immunoblotting. **(E)** Dose–response experiments to indicated concentrations of HGF in wt and MET∆14 cells. H3 was used as a loading control. Immunoblot pictures are representative of three independent experiments. Source data are available for this figure.

The *MET* oncogene is mutated in multiple cancer types, highlighting its critical role in cancer progression and invasion (for review, see Graveel et al [2013] and Comoglio et al [2018]). High throughput sequencing technologies have enabled the discovery of hotspot mutations in *MET*, opening new avenues for next-generation targeted therapies (Comoglio et al, 2018; Guo et al, 2020). We have used the publicly available MSK-IMPACT dataset (Zehir et al, 2017) and found that the splicing site mutations flanking exon 14 of the *MET* gene are by far the most frequent mutations observed in cancer patients (Fig 1A). Uncommonly for a receptor tyrosine kinase (e.g., EGFR, BCL-ABL, FGFR, and SRC in cancer, for review see Du and Lovly [2018]), very few patients displayed mutations in the kinase domain or regulatory regions (S985, Y1003, Y1234, Y1235, etc.). In line with these observations, studies targeting patients with MET∆14 are in progress (Paik et al, 2015, 2020; Engstrom et al, 2017; Klempner et al, 2017; Hu et al, 2018; Landi et al, 2019; Moro-Sibilot et al, 2019; Drilon et al, 2020; Wolf et al, 2020), but the clinical response was curiously restricted to a fraction of patients displaying Δ14 phenotypes (Salgia et al, 2020). These observations reflect the critical importance of the juxtamembrane domain encoded by exon 14 and urge us to explore MET∆14-driven oncogenicity. Mirroring the complexity of cancer multiple alterations may coexist, including *MET* gene amplification in addition to exon 14 “skipping” (Fig 1B). Thus, the common belief suggesting that MET∆14 is constitutively active may be due to the confounding effect of *MET* gene amplification or other activating mutations (Cortot et al, 2017; Descarpentries et al, 2018).

**Figure S1.**
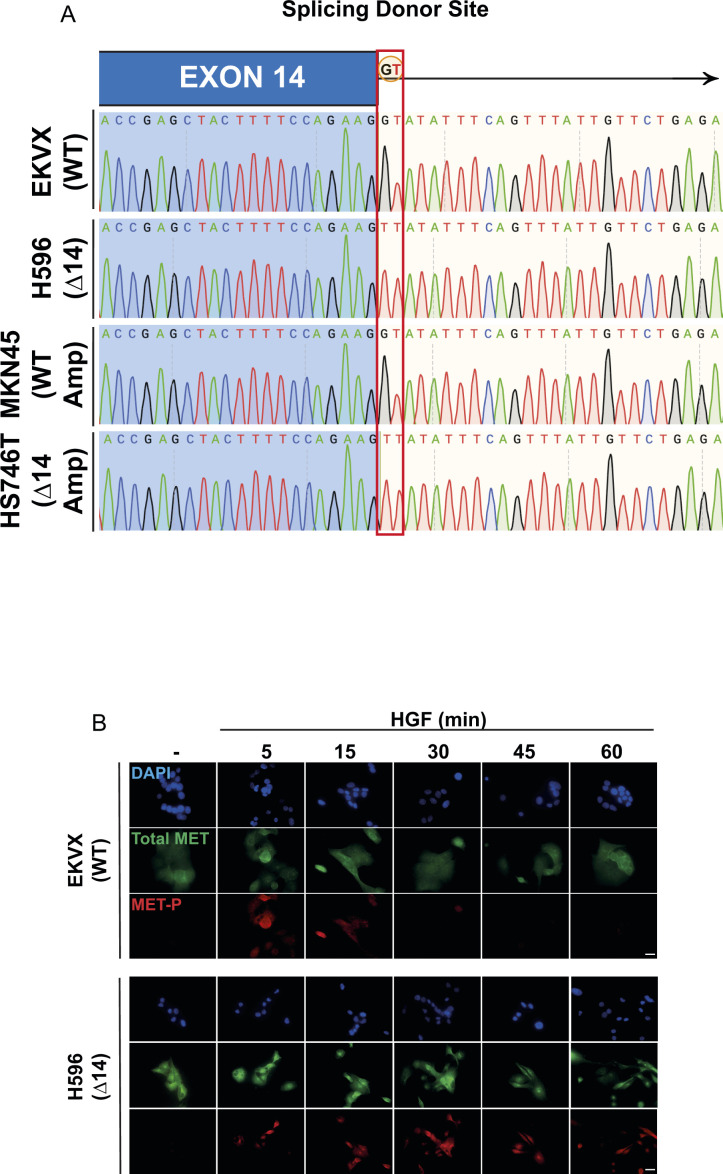
MET∆14 activation requires hepatocyte growth factor. **(A)** Sanger sequencing of MET gene from genomic DNA. G > T transversion in the splicing donor site leading to exon 14 skipping has been highlighted. **(B)** Kinetics of activation of WT MET and MET∆14 after the stated time of hepatocyte growth factor treatment, assessed by immunofluorescence. Scale bar = 25 μm. Results are representative pictures of N > 3 independent experiments.

To challenge the postulate of MET∆14 being constitutively active–as opposed to *MET* amplification or established activating mutations–we have tested MET tyrosine phosphorylation, on steady-state, in NCI-H596 cells (lung adenosquamous carcinoma cells, expressing MET∆14 [Ma et al, 2003]), EKVX cells (lung adenocarcinoma cells, expressing wt MET), HS746T (gastric adenocarcinoma cells, MET∆14 amplification), and MKN45 (gastric adenocarcinoma cells, wt MET amplification). For sake of clarity, the cells are therein denotated, respectively, as ∆14, WT, ∆14_Amp, and WT_Amp. Notably, NCI-H596 cells express only the mutated allele (Fig S1A). Strikingly, in the native state, MET∆14 was not phosphorylated (active) but depended on the presence of HGF, similar to cells expressing wt MET (Fig 1C). By contrast, cells harbouring *MET* gene amplification, either wt or ∆14, showed constitutive activation, as expected (for review, see Hong et al [2021]). These surprising results encouraged us to study the HGF-dependent activation of MET∆14 further. Immunoblot (Fig 1C) and immunofluorescence (Fig S1B) experiments confirmed the dependence of MET∆14 to HGF. We also noticed that HGF stimulation resulted in more sustained MET∆14 activation than wt MET. Moreover, compared with the levels of MET wt protein decaying over time after HGF stimulation as reported before (Vigna et al, 1999; Kong-Beltran et al, 2006), MET∆14 resisted this down-regulation (Fig 1D), suggesting a longer half-life, possibly because of the loss of Y1003, responsible of c-CBL binding and MET degradation (Peschard et al, 2001). Dose–response experiments elicited that ∆14 was activated in the presence of lower HGF concentrations than wt (Fig 1E). These observations established altered kinetics and HGF affinity of MET∆14 compared with its wt counterpart.

**Figure 2.**
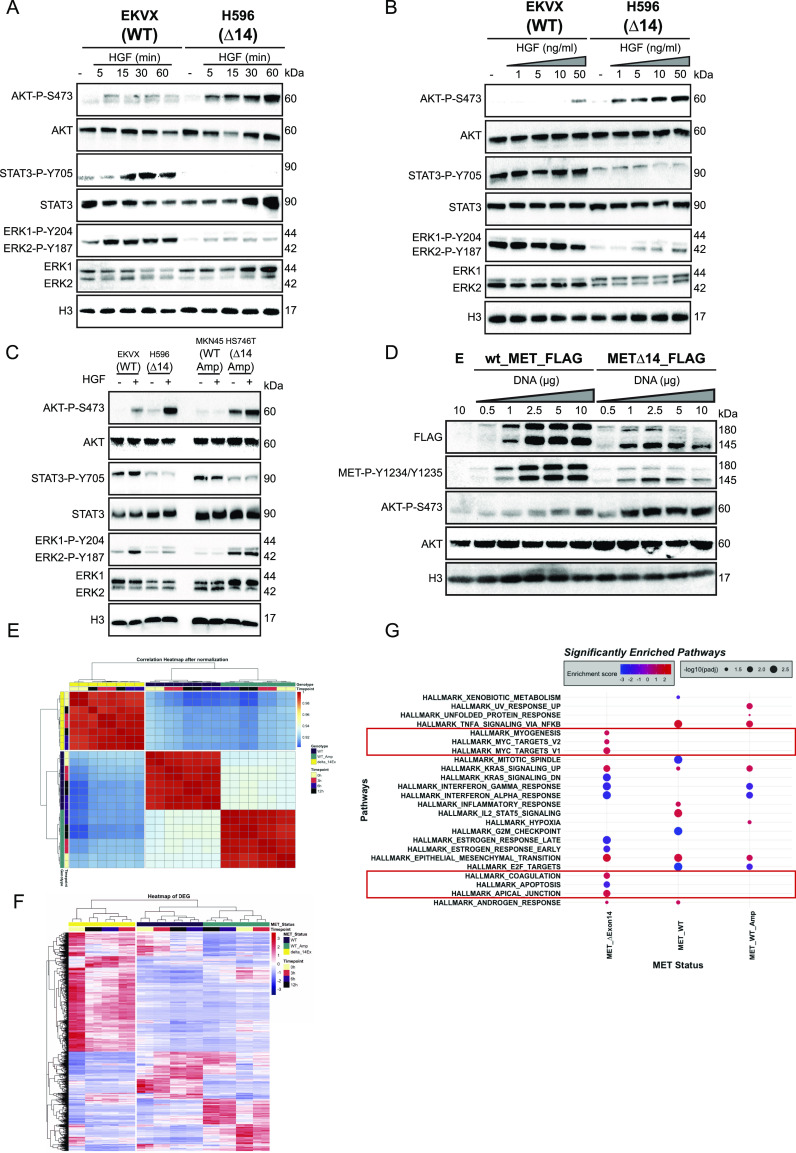
Hepatocyte growth factor (HGF)-induced MET∆14 activation displays singular pro-oncogenic features. **(A, B)** Immunoblot exposing the phosphorylation status of known primary downstream signal transducer associated with HGF/MET pathway. **(A, B)** Cells expressing either wt or MET∆14 were stimulated with 50 ng/ml of HGF during indicated times (A) or with indicated concentrations of HGF during 15 min (B). **(C)** Activation status of MET downstream transducers was assessed by immunoblotting in cells expressing wt, MET∆14, amplified wt MET, or amplified MET∆14 treated with ± HGF. H3 was used as a loading control. Immunoblot pictures are representative of three independent experiments. **(D)** TOV112D cells were transfected with either pCDNA-3xFlag (empty vector, E), pCDNA-wt_MET-3xFlag, or pCDNA-MET∆14-3xFlag. Immunoblots were performed 48 h post-transfection. **(E)** Cells were treated with HGF for 0, 3, 6, or 12 h. Experiments were performed in biological duplicates. After sequencing, differential gene expression analyses were conducted using the R package DESeq2 version 1.32.0 with the formula ∼Cell_line + time_of_HGF_treatment. The correlation heatmap was plotted using the pheatmap package. **(F)** Heatmap of top 500 differentially expressed genes after HGF treatment. **(G)** Gene set enrichment analyses on the genes whose expression values are significantly affected by HGF treatment.

**Figure S2.**
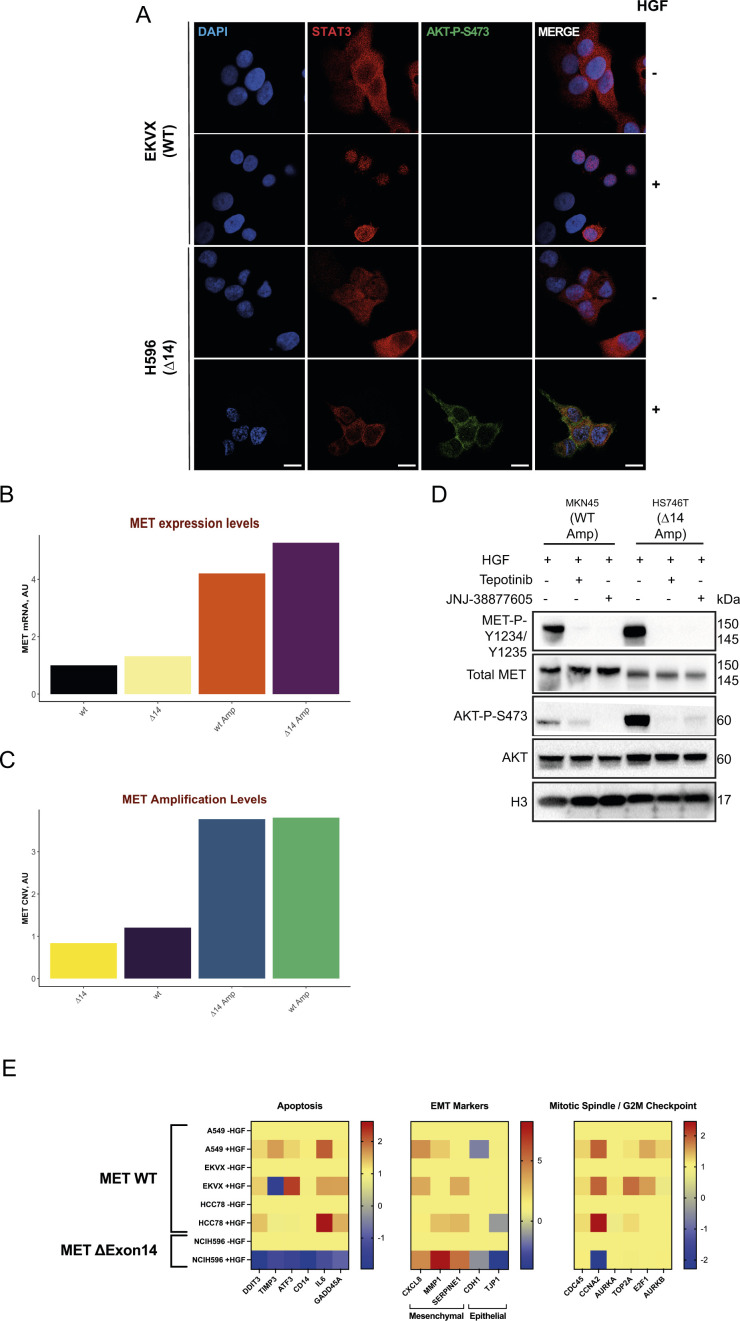
Hepatocyte growth factor-induced MET∆14 activation displays singular pro-oncogenic features. **(A)** Representative pictures showing STAT3 localisation (red) and AKT phosphorylation (green) in WT or MET∆14 expressing cells treated with ± hepatocyte growth factor for 15 min. Images are acquired with Leica SP5 confocal microscope. Scale bar = 12 μm. **(B)** MET mRNA expression levels in indicated cell lines at steady-state. **(C)** Copy number variation analysis of the MET gene. **(D)** Immunoblot analysis of indicated proteins in cells treated with indicated specific MET inhibitors. H3 was used as a loading control. **(E)** RT-qPCR analyses on multiple cell lines expressing WT MET compared with MET∆14.

To investigate the effect of long-lasting activation of MET∆14, we analysed the phosphorylation status of known downstream signal transducers such as ERK1/2 (for the RAS pathway), AKT (for the PI3K pathway), and STAT3 (Fig 2A and B) (Ponzetto et al, 1993, 1994; Fixman et al, 1996; Boccaccio et al, 1998). Intriguingly, we found that HGF-stimulated MET∆14 activation led to a robust AKT phosphorylation, a less-pronounced ERK1/2 phosphorylation and undetectable STAT3 activation compared with wt MET (Figs 2A and B and S2A). To rule out the possibility that the strong AKT activation, the weak of ERK1/2 phosphorylation, and the less-pronounced STAT3 phosphorylation were cell line-specific features—that is, unrelated to exon14 deletion—we have stimulated cancer cells expressing either wt, ∆14, amplified wt, or amplified MET∆14 with HGF (Fig 2C). The cells expressing wt and MET∆14 displayed similar MET transcript levels (Fig S2B), whereas *MET* gene amplification status of wt or ∆14 were comparable (Fig S2C). Strikingly, AKT phosphorylation was restricted to cells expressing MET∆14 regardless of their amplified status. On the other hand, cells expressing the wt copy of *MET*—and not *MET∆14* displayed STAT3 and ERK1/2 (MAPK) activation. Moreover, transfection of TOV112D cells—known not to express endogenous MET—with either wt MET-Flag or MET∆14-flag vectors further confirmed the ∆14-specific activation of the AKT pathway (Fig 2D). These results suggest that MET∆14 induces a robust and selective AKT activation at the cost of STAT3 and ERK1/2 phosphorylation. The MET-dependence of AKT phosphorylation in MET∆14-amplified cells was further demonstrated by hampering activation of AKT in HGF-stimulated cells treated with specific MET inhibitors (Fig S2D). AKT activation is specific to MET∆14 and cannot be explained by an overactive receptor because it has not been observed in wt MET–amplified cells. The facilitated interaction between p85 and MET∆14 was previously reported and can justify the strong AKT phosphorylation in ∆14 cells treated with HGF (Lee & Yamada, 1995). Therefore, HGF-activated MET∆14 acts as a conditional gain-of-function mutation, with the ensuing AKT activation potentially giving rise to a selective survival advantage to cancer cells expressing MET∆14.

To deepen the analysis of pathways influenced by HGF stimulation, we performed transcriptomic analysis in cells expressing either wt MET, amplified wt MET, or MET∆14, stimulated for the indicated times with HGF. Strikingly, unsupervised analysis of the transcriptional response to HGF induced by MET∆14 formed a gene cluster distinct from those produced by either naïve or amplified wt MET (Fig 2E). The heatmap of the 500 most differentially expressed genes after HGF stimulation displayed a distinctive transcriptomic profile in MET∆14 (Fig 2F). Gene set enrichment analysis was then performed to compare hallmark gene sets disturbed by HGF. Notably, six pathways were exclusively enriched in cells expressing MET∆14: myogenesis (epithelial–mesenchymal transition [EMT]–like pathway), MYC targets V1 and V2, apoptosis, coagulation, and apical junction (Fig 2G). To exclude the possibility of a cell line-rather than MET∆14-specific effect, qRT-PCR was performed in three genetically diverse *MET* wt cells (based on the CCLE dataset [Ghandi et al, 2019]). Regardless of genetic background, the studied MET wt cells displayed a similar pattern of transcriptional activation for the selected genes, including EMT, mitotic spindle, and apoptosis upon HGF activation, which was distinct from that observed in MET∆14 cells. Specifically, HGF induced a substantial down-regulation of apoptosis-related genes in the latter. EMT genes were more substantially affected (i.e., mesenchymal markers up-regulated, epithelial markers downregulated); mitotic spindle-associated genes were not impaired (Fig S2E).

Collectively, these results suggest that in MET∆14-expressing cells, the PI3K/AKT pathway is selectively activated upon HGF stimulation. Accordingly, the transcriptomic profile altered by MET∆14 supports an HGF-dependent inhibition of apoptosis and enhanced survival and migration capabilities.

**Figure 3.**
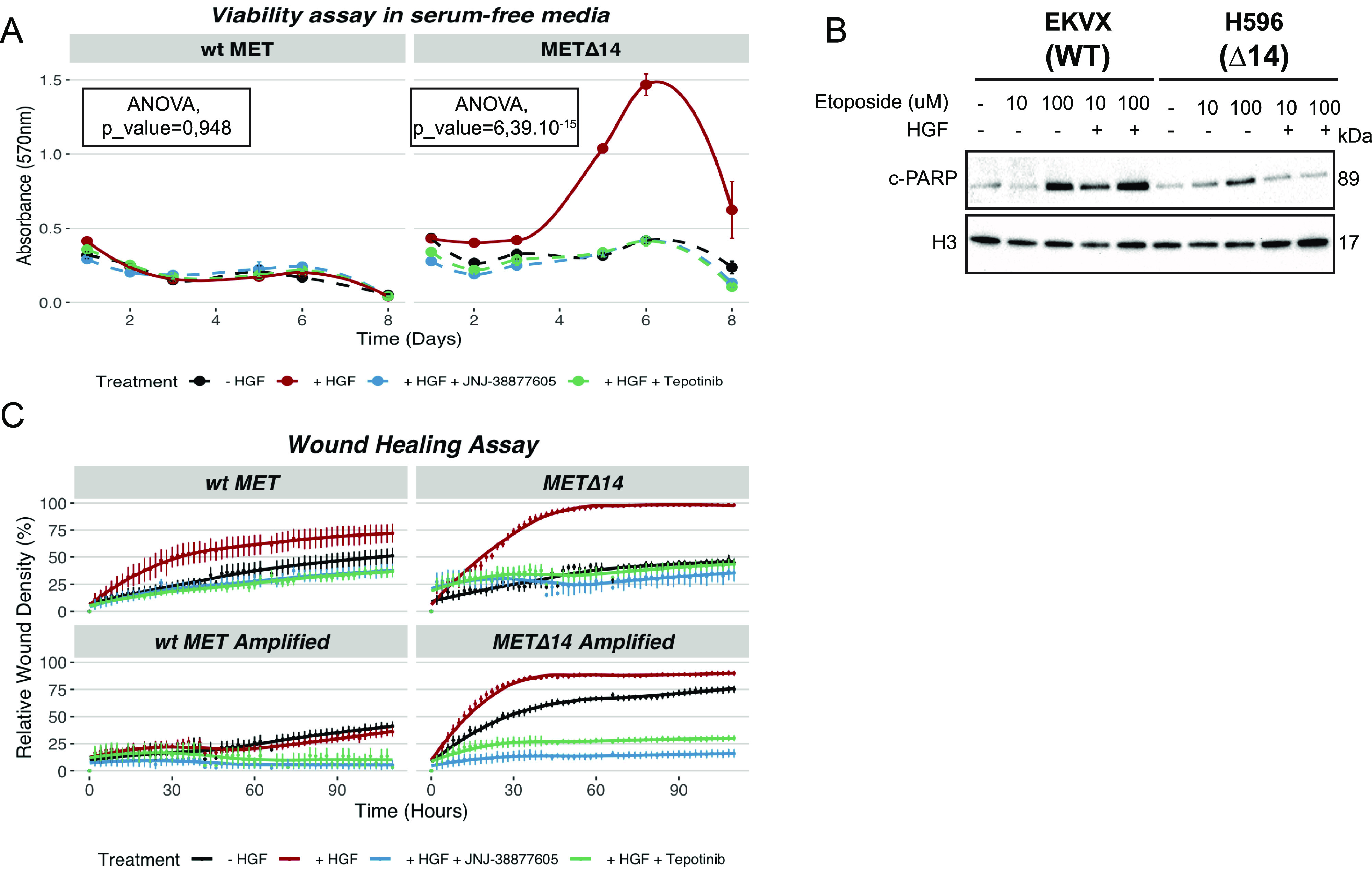
Hepatocyte growth factor (HGF)–induced MET∆14 activation enhances viability, confers resistance to apoptosis, and increases the migration potential of cancer cells. **(A)** MTT viability assay in cells expressing wt MET or MET∆14 ± HGF ± MET inhibitors for the indicated time. Results are presented as absorbance values at 570 nm ± SEM (N = 6/condition). **(B)** Immunoblot to visualise c-PARP1 in cells treated with ± HGF ± etoposide for 12 h. H3 was used as a loading control. **(C)** Quantification of the relative wound density in cells expressing wt MET, MET∆14, wt MET–amplified, or MET∆14-amplified. Results are the spatial cell density in the wound area relative to the spatial cell density outside the wound area at every time point ± SEM (N = 6/condition).

**Figure S3.**
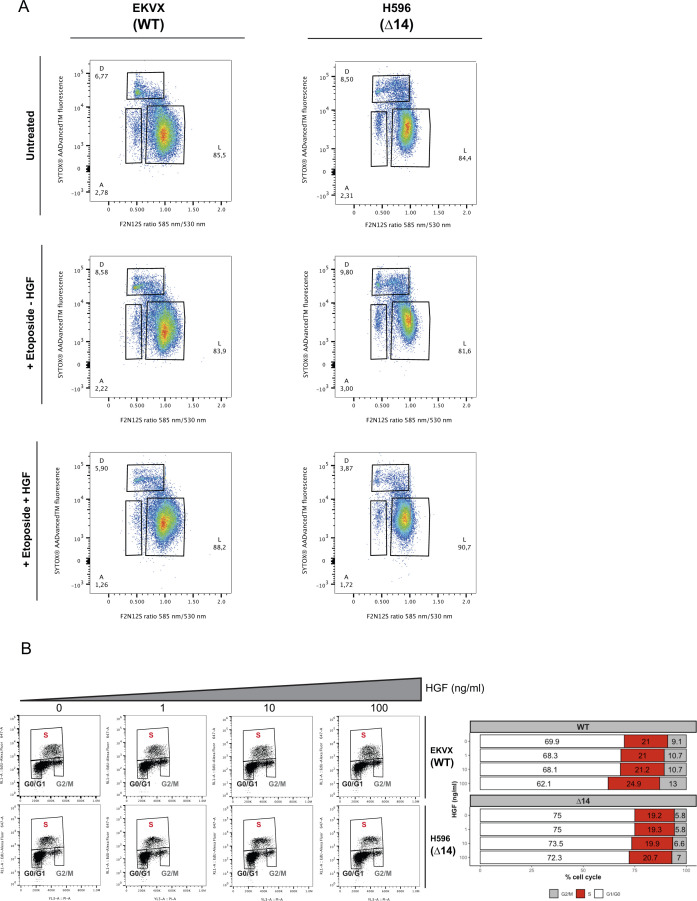
Hepatocyte growth factor (HGF)–induced MET∆14 activation enhances viability, confers resistance to apoptosis, and increases the migration potential of cancer cells. **(A)** Flow cytometry analyses of membrane asymmetry (F2N12S) and DNA labelling (SYTOX AADvancedTM) in non-fixed cells treated with ± HGF ± etoposide for 12 h. **(B)** EdU/PI experiment in cells expressing WT MET or MET∆14 treated with indicated concentrations of HGF for 12 h. Results are shown as the percentage of cells in each cell cycle phase after flow cytometry analyses.

We have then directly tested the aforementioned assumptions. First, we performed a viability assay in the absence of serum and showed that MET∆14—and not wt MET–induced serum-independent growth (Fig 3A). Notably, the viability was compromised by the specific MET inhibitors tepotinib (Paik et al, 2020) and JNJ-38877605 (De Bacco et al, 2011). Second, we have treated cells expressing either wt or MET∆14 with etoposide, a commonly used genotoxic agent in lung cancer patients (Slevin et al, 1989). In the presence of HGF, MET∆14 cells were more effectively protected from etoposide-induced apoptosis than WT cells (Figs 3B and S3A). Third, to test how MET∆14 may promote cellular migration, we have measured wound healing induced by HGF (Fig 3C and Videos 1–Videos 16 online). Strikingly, MET∆14 completely covered the wound after 30 h whereas only half of the wound was covered in wt cells. In addition, faster wound healing was observed in MET∆14-amplified cells compared with MET_wt-amplified cells. In ∆14-amplified cells, the wound was healed in a ligand-independent manner. By contrast, cells expressing wt MET (amplified or not) never wholly covered the wound. In all cell lines studied, specific MET inhibitors abolished the process. These results suggest that faster wound healing is a particular feature conferred by MET∆14.

Video 1 Download video Representative wound healing videos in cells expressing wt MET, MET∆14, wt MET–amplified, or MET∆14-amplified. Cells were grown in the presence of the indicated drugs. Videos were recorded every 2 h for 5 d (N = 6/condition).

Video 2 Download video Representative wound healing videos in cells expressing wt MET, MET∆14, wt MET–amplified, or MET∆14-amplified. Cells were grown in the presence of the indicated drugs. Videos were recorded every 2 h for 5 d (N = 6/condition).

Video 3 Download video Representative wound healing videos in cells expressing wt MET, MET∆14, wt MET–amplified, or MET∆14-amplified. Cells were grown in the presence of the indicated drugs. Videos were recorded every 2 h for 5 d (N = 6/condition).

Video 4 Download video Representative wound healing videos in cells expressing wt MET, MET∆14, wt MET–amplified, or MET∆14-amplified. Cells were grown in the presence of the indicated drugs. Videos were recorded every 2 h for 5 d (N = 6/condition).

Video 5 Download video Representative wound healing videos in cells expressing wt MET, MET∆14, wt MET–amplified, or MET∆14-amplified. Cells were grown in the presence of the indicated drugs. Videos were recorded every 2 h for 5 d (N = 6/condition).

Video 6 Download video Representative wound healing videos in cells expressing wt MET, MET∆14, wt MET–amplified, or MET∆14-amplified. Cells were grown in the presence of the indicated drugs. Videos were recorded every 2 h for 5 d (N = 6/condition).

Video 7 Download video Representative wound healing videos in cells expressing wt MET, MET∆14, wt MET–amplified, or MET∆14-amplified. Cells were grown in the presence of the indicated drugs. Videos were recorded every 2 h for 5 d (N = 6/condition).

Video 8 Download video Representative wound healing videos in cells expressing wt MET, MET∆14, wt MET–amplified, or MET∆14-amplified. Cells were grown in the presence of the indicated drugs. Videos were recorded every 2 h for 5 d (N = 6/condition).

Video 9 Download video Representative wound healing videos in cells expressing wt MET, MET∆14, wt MET–amplified, or MET∆14-amplified. Cells were grown in the presence of the indicated drugs. Videos were recorded every 2 h for 5 d (N = 6/condition).

Video 10 Download video Representative wound healing videos in cells expressing wt MET, MET∆14, wt MET–amplified, or MET∆14-amplified. Cells were grown in the presence of the indicated drugs. Videos were recorded every 2 h for 5 d (N = 6/condition).

Video 11 Download video Representative wound healing videos in cells expressing wt MET, MET∆14, wt MET–amplified, or MET∆14-amplified. Cells were grown in the presence of the indicated drugs. Videos were recorded every 2 h for 5 d (N = 6/condition).

Video 12 Download video Representative wound healing videos in cells expressing wt MET, MET∆14, wt MET–amplified, or MET∆14-amplified. Cells were grown in the presence of the indicated drugs. Videos were recorded every 2 h for 5 d (N = 6/condition).

Video 13 Download video Representative wound healing videos in cells expressing wt MET, MET∆14, wt MET–amplified, or MET∆14-amplified. Cells were grown in the presence of the indicated drugs. Videos were recorded every 2 h for 5 d (N = 6/condition).

Video 14 Download video Representative wound healing videos in cells expressing wt MET, MET∆14, wt MET–amplified, or MET∆14-amplified. Cells were grown in the presence of the indicated drugs. Videos were recorded every 2 h for 5 d (N = 6/condition).

Video 15 Download video Representative wound healing videos in cells expressing wt MET, MET∆14, wt MET–amplified, or MET∆14-amplified. Cells were grown in the presence of the indicated drugs. Videos were recorded every 2 h for 5 d (N = 6/condition).

Video 16 Download video Representative wound healing videos in cells expressing wt MET, MET∆14, wt MET–amplified, or MET∆14-amplified. Cells were grown in the presence of the indicated drugs. Videos were recorded every 2 h for 5 d (N = 6/condition).

Increased viability and faster wound healing may result from MET∆14-induced cell proliferation. To explore a potential MET∆14-induced cell proliferation, cell cycle progression was assessed by EdU/PI labelling in MET∆14 and wt cells in concert with increasing concentrations of HGF (Fig S3B). In line with previous reports (Anastasi et al, 1997), HGF induced a modest concentration-dependent increase in the number of cells in the S phase in wt cells. Surprisingly, HGF did not increase the percentage of cells in the S phase in MET∆14. We conclude from these observations that the potent HGF-dependent wound healing response and the increased viability conferred by the HGF/MET∆14 axis are due to enhanced cell migration and survival rather than increased proliferation.

Thus, MET∆14 seems to act as a ligand-dependent gain-of-function enhancing cancer cell survival and migration capabilities. When activated by HGF released by the tumour microenvironment (e.g., by cancer-associated fibroblasts), MET∆14 activates the PI3K/AKT pathway without inducing STAT3 (differentiation) or MAPK (proliferation) phosphorylation. Thus, cancer cells increase their probability of survival within the tumour pseudo-organ, which possesses a myriad of unfavourable conditions, including shortage of nutrients, hypoxia, radiotherapy, and chemotherapy. We hypothesize that an enhanced migratory potential conferred by the MET∆14/PI3K/AKT axis allows cells to escape this hostile environment and form distal metastasis. Consequently, cells harbouring MET∆14 may choose to “fly” instead of “fight” locally by proliferation. Besides, migrating instead of dividing may constitute a shrewd strategy to escape an adverse local micromilieu for cell survival because most cancer drugs are designed to destroy actively dividing cells.

MET∆14 appears to be central to tumour cell survival. Hence in-depth understanding of the advantages it provides to cancer cells may be therapeutically exploited to target their vulnerability. However, the response to MET inhibitors is observed only in a fraction of ∆14 patients. Our present results suggest that the resistance to targeted therapy may result from the absence of HGF in the tumour microenvironment or from activating mutations in the PI3K/AKT axis. Interestingly, the latter have been reported to coexist with MET exon 14 skipping in 14.2% of lung cancer patients (Jamme et al, 2020; Rotow et al, 2020).

Overall, our findings enlighten the mechanism of HGF-dependent MET∆14 activation and show that conditions other than mutations of the splicing site of exon 14 should be considered. Further preclinical and clinical work, including endogenous HGF levels and the mutational status PI3K/AKT axis, is required to justify the stratification of patients for clinical trials.

## Materials and Methods

### Reagents

Tepotinib (S7067), JNJ-38877605 (S1114), and etoposide (S1225) were purchased from Selleckchem. Recombinant HGF (294-HGN-025/CF) was bought from R&D Systems.

### Plasmids

3XFlag Tag was inserted into the pCDNA3.1+ vector between the HindIII and BamHI restriction sites. wt_MET or MET∆14 cDNA were inserted into pCDNA-3XFlag vector between NheI and HindIII restriction sites. Plasmids used in this study are available on Addgene with Addgene IDs 182494, 182495, and 182496.

### Cell culture

NCI-H596, HS746T, and TOV112D cells were purchased from ATCC. EKVX and MKN45 were obtained from the Cell Culture facility of Istituto Fondazione di Oncologia Molecolare (IFOM). Cells were cultured according to manufacturer’s instructions and regularly verified for the absence of mycoplasma contamination. Experiments were performed before passage number 20. For HGF treatments, cells were serum-deprived for 24 h and treated with indicated time and concentrations of HGF. TOV112D cells were transfected with indicated quantity of DNA in 6-cm-diameter dishes using Lipofectamine2000 reagent (Cat. no. 11668019; Thermo Fisher Scientific) according to the manufacturer’s instructions.

### MET alterations

Genomic DNA was extracted from cells using phenol/chloroform extraction. DNA was treated with RNase, and PCR reaction was performed using primers flanking the exon 14 of the *MET* gene (forward: 5′-GTCGTCGATTCTTGTGTGCTG-3′; reverse: 5′-GGGCTTCAACAGGTAAAAAATG-3′) with the Phusion High-Fidelity DNA Polymerase (M0530; New England Biolabs). The PCR product was sequenced by Sanger sequencing to assess *MET* mutational status. Besides, *MET* amplification status was evaluated using the MET Taqman probes (Hs04957390_cn and Hs05011082_cn). TERT (Hs02088500_cn) and RNAseP (Hs05182199_cn) genes were used as internal control for the assay.

### RNA extraction

Total RNA was extracted using QIAGEN RNeasy Mini Kit (Cat. no. 74106). For RNAseq experiments, quality assessment and library preparations were carried out as previously described (Nosi et al, 2021). Reads were trimmed to remove adapters sequences and mapped using STAR on ENSEMBL hg38 human genome assembly.

### Immunoblotting

Cells were lysed in Laemmli buffer as previously described (Modica et al, 2021). Proteins were resolved in pre-casted polyacrylamide gels (Thermo Fisher Scientific) and blotted against antibodies listed in Table S1.

Table S1 List of antibodies used in the study.

### Immunofluorescence

Cells were plated in chamber slides (Thermo Fisher Scientific, Nunc Lab-Tek II Chamber Slide System, 154461 PK). Immunostainings were performed as previously described (Czibik et al, 2021). Antibodies are listed in Table S1. Images were acquired with the Leica TCS SP5 microscope.

### Viability assay

Cells were plated in 96-well plates at a density of 5,000 cells/well. After 24-h starvation, the cells were treated with ± HGF ± indicated MET inhibitors. Cell viability was assessed using CellTiter 96 Non-Radioactive Cell Proliferation Assay (G4100; Promega) every day for 80 d. The culture medium was renewed every 3 d.

### Wound healing assay

Cells were plated in 96-well plates at a density of 40,000 cells/well. After 24-h starvation, the cells were treated with ± HGF ± indicated MET inhibitors. After the wound generation, the relative wound density was measured by Incucyte (Sartorius) according to the manufacturer’s instructions.

### Apoptosis assay

Cells were treated for 18 h with 10 μM etoposide ± HGF. Apoptosis was assessed by immunoblotting using antibodies against cleaved PARP.

### Code availability

Data were analysed using R version 4.1.2. Codes are available at: https://github.com/Altintas-D/MET-14-promotes-a-ligand-dependent-AKT-driven-invasive-growth.

### qRT-PCR

1 μg of total RNA was reverse transcribed using Superscript III Reverse Transcriptase (Cat. no. 18080; Thermo Fisher Scientific) and relative gene expression levels were assessed using Taqman probes (Table S2). TBP and 18S were used as normalisation genes.

Table S2 List of TaqMan probes used in the study.

### Apoptosis assay

Cells were treated for 18 h with 10 μM etoposide ± HGF. Apoptosis was assessed by flow cytometry using the Violet Ratiometric Membrane Asymmetry Probe/Dead Cell Apoptosis Kit (Cat. no. A35137; Thermo Fisher Scientific).

### Cell cycle analysis

Cell proliferation was assessed by measuring EdU incorporation by flow cytometry. Briefly, cells were treated with indicated concentrations of HGF. After 2 h of EdU treatment, cells were pelleted and fixed, labelled with anti-EdU-Alexa Fluor647 and propidium iodide (DNA) using the Click-iT EdU Flow Cytometry Assay kit (C10424; Thermo Fisher Scientific).

## Data Availability

The RNAseq data from this publication have been deposited to Gene Expression Omnibus database (https://www.ncbi.nlm.nih.gov/geo/) and assigned the identifier GSE194382.

## Supplementary Material

Reviewer comments

## References

[bib1] Anastasi S, Giordano S, Sthandier O, Gambarotta G, Maione R, Comoglio P, Amati P (1997) A natural hepatocyte growth factor/scatter factor autocrine loop in myoblast cells and the effect of the constitutive Met kinase activation on myogenic differentiation. J Cell Biol 137: 1057–1068. 10.1083/jcb.137.5.1057 9166406 PMC2136220

[bib2] Boccaccio C, Andò M, Tamagnone L, Bardelli A, Michieli P, Battistini C, Comoglio PM (1998) Induction of epithelial tubules by growth factor HGF depends on the STAT pathway. Nature 391: 285–288. 10.1038/34657 9440692

[bib3] Boccaccio C, Comoglio PM (2006) Invasive growth: A MET-driven genetic programme for cancer and stem cells. Nat Rev Cancer 6: 637–645. 10.1038/nrc1912 16862193

[bib4] Comoglio PM, Trusolino L, Boccaccio C (2018) Known and novel roles of the MET oncogene in cancer: A coherent approach to targeted therapy. Nat Rev Cancer 18: 341–358. 10.1038/s41568-018-0002-y 29674709

[bib5] Cortot AB, Kherrouche Z, Descarpentries C, Wislez M, Baldacci S, Furlan A, Tulasne D (2017) Exon 14 deleted MET receptor as a new biomarker and target in cancers. J Natl Cancer Inst 109: 262–273. 10.1093/jnci/djw262 28376232

[bib6] Czibik G, Mezdari Z, Murat Altintas D, Bréhat J, Pini M, d’Humières T, Delmont T, Radu C, Breau M, Liang H, (2021) Dysregulated phenylalanine catabolism plays a key role in the trajectory of cardiac aging. Circulation 144: 559–574. 10.1161/CIRCULATIONAHA.121.054204 34162223

[bib7] De Bacco F, Luraghi P, Medico E, Reato G, Girolami F, Perera T, Gabriele P, Comoglio PM, Boccaccio C (2011) Induction of MET by ionizing radiation and its role in radioresistance and invasive growth of cancer. J Natl Cancer Inst 103: 645–661. 10.1093/jnci/djr093 21464397

[bib8] de Luca A, Arena N, Sena LM, Medico E (1999) Met overexpression confers HGF-dependent invasive phenotype to human thyroid carcinoma cells in vitro. J Cell Physiol 180: 365–371. 10.1002/(SICI)1097-4652(199909)180:3<365::AID-JCP7>3.0.CO;2-B 10430176

[bib9] Descarpentries C, Leprêtre F, Escande F, Kherrouche Z, Figeac M, Sebda S, Baldacci S, Grégoire V, Jamme P, Copin MC, (2018) Optimization of routine testing for MET exon 14 splice site mutations in NSCLC patients. J Thorac Oncol 13: 1873–1883. 10.1016/j.jtho.2018.08.2023 30195702

[bib10] Drilon A, Clark JW, Weiss J, Ou SI, Camidge DR, Solomon BJ, Otterson GA, Villaruz LC, Riely GJ, Heist RS, (2020) Antitumor activity of crizotinib in lung cancers harboring a MET exon 14 alteration. Nat Med 26: 47–51. 10.1038/s41591-019-0716-8 31932802 PMC8500676

[bib11] Du Z, Lovly CM (2018) Mechanisms of receptor tyrosine kinase activation in cancer. Mol Cancer 17: 58. 10.1186/s12943-018-0782-4 29455648 PMC5817791

[bib12] Engstrom LD, Aranda R, Lee M, Tovar EA, Essenburg CJ, Madaj Z, Chiang H, Briere D, Hallin J, Lopez-Casas PP, (2017) Glesatinib exhibits antitumor activity in lung cancer models and patients harboring MET exon 14 mutations and overcomes mutation-mediated resistance to type I MET inhibitors in nonclinical models. Clin Cancer Res 23: 6661–6672. 10.1158/1078-0432.CCR-17-1192 28765324

[bib13] Fixman ED, Fournier TM, Kamikura DM, Naujokas MA, Park M (1996) Pathways downstream of Shc and Grb2 are required for cell transformation by the tpr-Met oncoprotein. J Biol Chem 271: 13116–13122. 10.1074/jbc.271.22.13116 8662733

[bib14] Fujino T, Suda K, Mitsudomi T (2021) Lung cancer with MET exon 14 skipping mutation: Genetic feature, current treatments, and future challenges. Lung Cancer (Auckl) 12: 35–50. 10.2147/LCTT.S269307 34295201 PMC8290191

[bib15] Galimi F, Brizzi MF, Comoglio PM (1993) The hepatocyte growth factor and its receptor. Stem Cells 11: 22–30. 10.1002/stem.5530110805 8401259

[bib16] Ghandi M, Huang FW, Jané-Valbuena J, Kryukov GV, Lo CC, McDonald ER 3rd, Barretina J, Gelfand ET, Bielski CM, Li H, (2019) Next-generation characterization of the cancer cell line encyclopedia. Nature 569: 503–508. 10.1038/s41586-019-1186-3 31068700 PMC6697103

[bib17] Graveel CR, Tolbert D, Vande Woude GF (2013) MET: A critical player in tumorigenesis and therapeutic target. Cold Spring Harb Perspect Biol 5: a009209. 10.1101/cshperspect.a009209 23818496 PMC3685898

[bib18] Guo R, Luo J, Chang J, Rekhtman N, Arcila M, Drilon A (2020) MET-dependent solid tumours - molecular diagnosis and targeted therapy. Nat Rev Clin Oncol 17: 569–587. 10.1038/s41571-020-0377-z 32514147 PMC7478851

[bib19] Hong L, Zhang J, Heymach JV, Le X (2021) Current and future treatment options for MET exon 14 skipping alterations in non-small cell lung cancer. Ther Adv Med Oncol 13: 1758835921992976. 10.1177/1758835921992976 33643443 PMC7890719

[bib20] Hu H, Mu Q, Bao Z, Chen Y, Liu Y, Chen J, Wang K, Wang Z, Nam Y, Jiang B, (2018) Mutational landscape of secondary glioblastoma guides MET-targeted trial in brain tumor. Cell 175: 1665–1678.e18. 10.1016/j.cell.2018.09.038 30343896

[bib21] Jamme P, Fernandes M, Copin MC, Descarpentries C, Escande F, Morabito A, Grégoire V, Jamme M, Baldacci S, Tulasne D, (2020) Alterations in the PI3K pathway drive resistance to MET inhibitors in NSCLC harboring MET exon 14 skipping mutations. J Thorac Oncol 15: 741–751. 10.1016/j.jtho.2020.01.027 32169477

[bib22] Klempner SJ, Borghei A, Hakimian B, Ali SM, Ou SI (2017) Intracranial activity of cabozantinib in MET exon 14-positive NSCLC with brain metastases. J Thorac Oncol 12: 152–156. 10.1016/j.jtho.2016.09.127 27693535

[bib23] Kong-Beltran M, Seshagiri S, Zha J, Zhu W, Bhawe K, Mendoza N, Holcomb T, Pujara K, Stinson J, Fu L, (2006) Somatic mutations lead to an oncogenic deletion of met in lung cancer. Cancer Res 66: 283–289. 10.1158/0008-5472.CAN-05-2749 16397241

[bib24] Landi L, Chiari R, Tiseo M, D’Incà F, Dazzi C, Chella A, Delmonte A, Bonanno L, Giannarelli D, Cortinovis DL, (2019) Crizotinib in MET-deregulated or ROS1-rearranged pretreated non-small cell lung cancer (METROS): A phase II, prospective, multicenter, two-arms trial. Clin Cancer Res 25: 7312–7319. 10.1158/1078-0432.CCR-19-0994 31416808

[bib25] Lee CC, Yamada KM (1995) Alternatively spliced juxtamembrane domain of a tyrosine kinase receptor is a multifunctional regulatory site. Deletion alters cellular tyrosine phosphorylation pattern and facilitates binding of phosphatidylinositol-3-OH kinase to the hepatocyte growth factor receptor. J Biol Chem 270: 507–510. 10.1074/jbc.270.2.507 7822270

[bib26] Lee CC, Yamada KM (1994) Identification of a novel type of alternative splicing of a tyrosine kinase receptor. Juxtamembrane deletion of the c-met protein kinase C serine phosphorylation regulatory site. J Biol Chem 269: 19457–19461. 10.1016/s0021-9258(17)32190-7 7518457

[bib27] Lee JH, Gao CF, Lee CC, Kim MD, Vande Woude GF (2006) An alternatively spliced form of Met receptor is tumorigenic. Exp Mol Med 38: 565–573. 10.1038/emm.2006.66 17079873

[bib28] Ma PC, Kijima T, Maulik G, Fox EA, Sattler M, Griffin JD, Johnson BE, Salgia R (2003) c-MET mutational analysis in small cell lung cancer: Novel juxtamembrane domain mutations regulating cytoskeletal functions. Cancer Res 63: 6272–6281. 14559814

[bib29] Modica C, Basilico C, Chiriaco C, Borrelli N, Comoglio PM, Vigna E (2021) A receptor-antibody hybrid hampering MET-driven metastatic spread. J Exp Clin Cancer Res 40: 32. 10.1186/s13046-020-01822-5 33446252 PMC7807714

[bib30] Moro-Sibilot D, Cozic N, Pérol M, Mazières J, Otto J, Souquet PJ, Bahleda R, Wislez M, Zalcman G, Guibert SD, (2019) Crizotinib in c-MET- or ROS1-positive NSCLC: Results of the AcSé phase II trial. Ann Oncol 30: 1985–1991. 10.1093/annonc/mdz407 31584608

[bib31] Nosi V, Luca A, Milan M, Arigoni M, Benvenuti S, Cacchiarelli D, Cesana M, Riccardo S, Di Filippo L, Cordero F, (2021) MET exon 14 skipping: A case study for the detection of genetic variants in cancer driver genes by deep learning. Int J Mol Sci 22: 4217. 10.3390/ijms22084217 33921709 PMC8072630

[bib32] Paik PK, Drilon A, Fan PD, Yu H, Rekhtman N, Ginsberg MS, Borsu L, Schultz N, Berger MF, Rudin CM, (2015) Response to MET inhibitors in patients with stage IV lung adenocarcinomas harboring MET mutations causing exon 14 skipping. Cancer Discov 5: 842–849. 10.1158/2159-8290.CD-14-1467 25971939 PMC4658654

[bib33] Paik PK, Felip E, Veillon R, Sakai H, Cortot AB, Garassino MC, Mazieres J, Viteri S, Senellart H, Van Meerbeeck J, (2020) Tepotinib in non-small-cell lung cancer with MET exon 14 skipping mutations. N Engl J Med 383: 931–943. 10.1056/NEJMoa2004407 32469185 PMC8422679

[bib34] Peschard P, Fournier TM, Lamorte L, Naujokas MA, Band H, Langdon WY, Park M (2001) Mutation of the c-Cbl TKB domain binding site on the Met receptor tyrosine kinase converts it into a transforming protein. Mol Cell 8: 995–1004. 10.1016/s1097-2765(01)00378-1 11741535

[bib35] Ponzetto C, Bardelli A, Maina F, Longati P, Panayotou G, Dhand R, Waterfield MD, Comoglio PM (1993) A novel recognition motif for phosphatidylinositol 3-kinase binding mediates its association with the hepatocyte growth factor/scatter factor receptor. Mol Cell Biol 13: 4600–4608. 10.1128/mcb.13.8.4600 7687741 PMC360084

[bib36] Ponzetto C, Bardelli A, Zhen Z, Maina F, dalla Zonca P, Giordano S, Graziani A, Panayotou G, Comoglio PM (1994) A multifunctional docking site mediates signaling and transformation by the hepatocyte growth factor/scatter factor receptor family. Cell 77: 261–271. 10.1016/0092-8674(94)90318-2 7513258

[bib37] Reungwetwattana T, Ou SH (2015) MET exon 14 deletion (METex14): Finally, a frequent-enough actionable oncogenic driver mutation in non-small cell lung cancer to lead MET inhibitors out of “40 years of wilderness” and into a clear path of regulatory approval. Transl Lung Cancer Res 4: 820–824. 10.3978/j.issn.2218-6751.2015.12.03 26798595 PMC4700217

[bib38] Rotow JK, Gui P, Wu W, Raymond VM, Lanman RB, Kaye FJ, Peled N, Fece de la Cruz F, Nadres B, Corcoran RB, (2020) Co-occurring alterations in the RAS-MAPK pathway limit response to MET inhibitor treatment in MET exon 14 skipping mutation-positive lung cancer. Clin Cancer Res 26: 439–449. 10.1158/1078-0432.CCR-19-1667 31548343 PMC6980768

[bib39] Salgia R, Sattler M, Scheele J, Stroh C, Felip E (2020) The promise of selective MET inhibitors in non-small cell lung cancer with MET exon 14 skipping. Cancer Treat Rev 87: 102022. 10.1016/j.ctrv.2020.102022 32334240

[bib40] Slevin ML, Clark PI, Joel SP, Malik S, Osborne RJ, Gregory WM, Lowe DG, Reznek RH, Wrigley PF (1989) A randomized trial to evaluate the effect of schedule on the activity of etoposide in small-cell lung cancer. J Clin Oncol 7: 1333–1340. 10.1200/JCO.1989.7.9.1333 2549204

[bib41] Tan YH, Krishnaswamy S, Nandi S, Kanteti R, Vora S, Onel K, Hasina R, Lo FY, El-Hashani E, Cervantes G, (2010) CBL is frequently altered in lung cancers: Its relationship to mutations in MET and EGFR tyrosine kinases. PLoS One 5: e8972. 10.1371/journal.pone.0008972 20126411 PMC2813301

[bib42] Vigna E, Gramaglia D, Longati P, Bardelli A, Comoglio PM (1999) Loss of the exon encoding the juxtamembrane domain is essential for the oncogenic activation of TPR-MET. Oncogene 18: 4275–4281. 10.1038/sj.onc.1202791 10435641

[bib43] Villa-Moruzzi E, Puntoni F, Bardelli A, Vigna E, De Rosa S, Comoglio PM (1998) Protein tyrosine phosphatase PTP-S binds to the juxtamembrane region of the hepatocyte growth factor receptor Met. Biochem J 336: 235–239. 10.1042/bj3360235 9806906 PMC1219863

[bib44] Wolf J, Seto T, Han JY, Reguart N, Garon EB, Groen HJM, Tan DSW, Hida T, de Jonge M, Orlov SV, (2020) Capmatinib in MET exon 14-mutated or MET-amplified non-small-cell lung cancer. N Engl J Med 383: 944–957. 10.1056/NEJMoa2002787 32877583

[bib45] Zehir A, Benayed R, Shah RH, Syed A, Middha S, Kim HR, Srinivasan P, Gao J, Chakravarty D, Devlin SM, (2017) Mutational landscape of metastatic cancer revealed from prospective clinical sequencing of 10,000 patients. Nat Med 23: 703–713. 10.1038/nm.4333 28481359 PMC5461196

