# Supplementary figures and images for: MET∆14 promotes a ligand-dependent, AKT-driven invasive growth
Source: Life Sci Alliance. 2022 May 30;5(10):e202201409. doi: 10.26508/lsa.202201409 (PMC9152130; doi:10.26508/lsa.202201409)

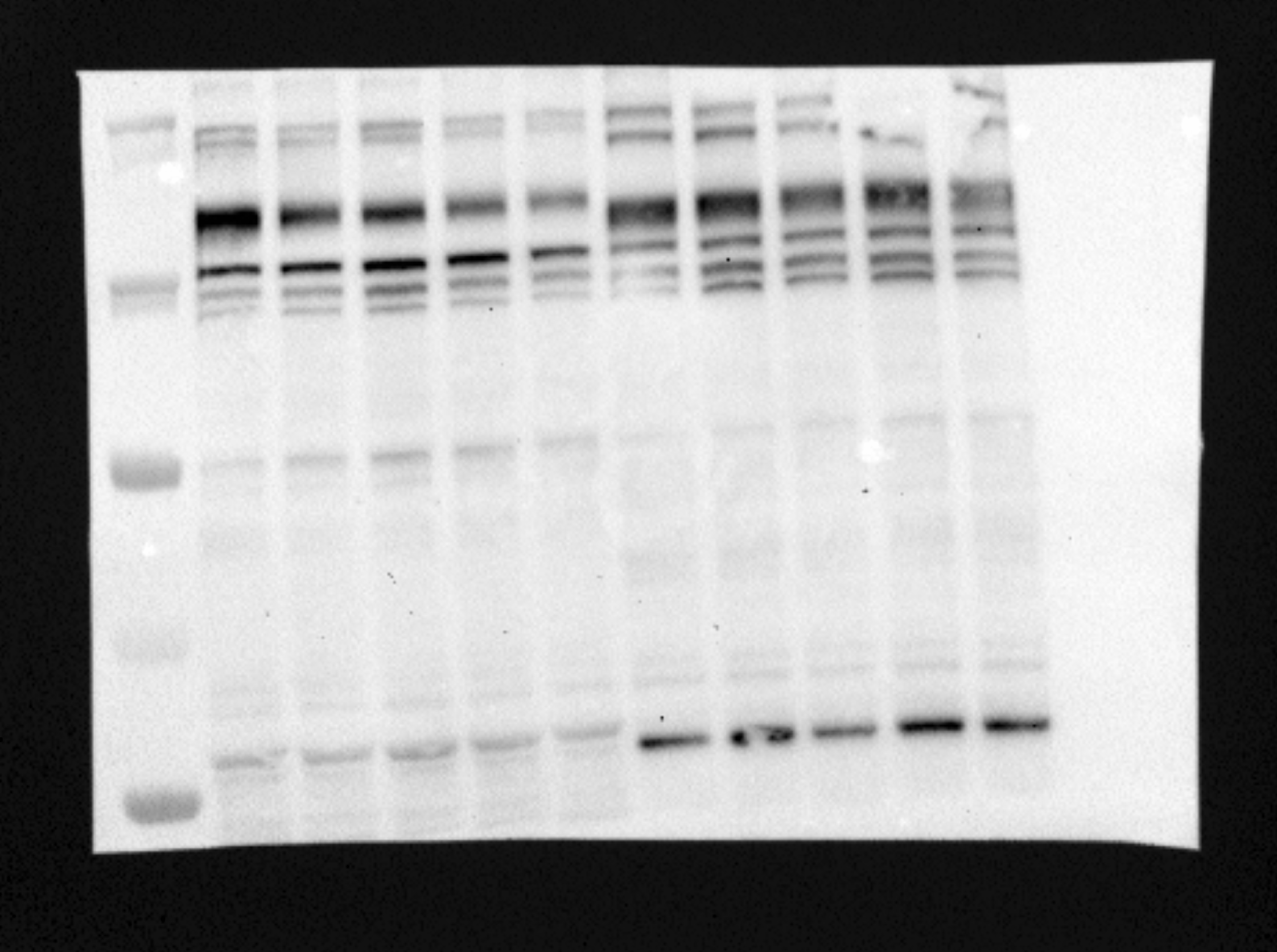

Supplement: Supplementary file 1 [file LSA-2022-01409_SdataF1.1.tif]

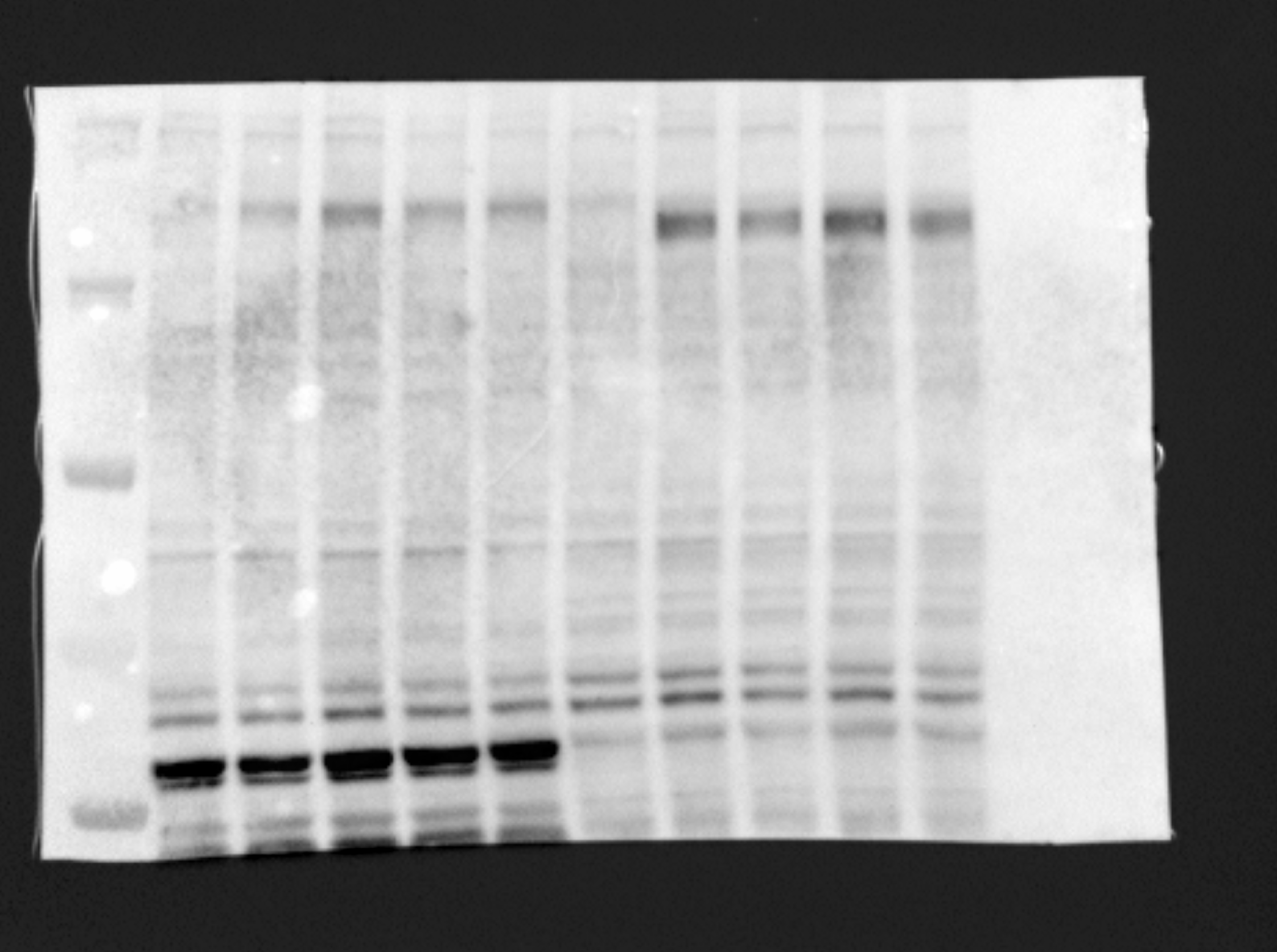

Supplement: Supplementary file 2 [file LSA-2022-01409_SdataF1.2.tif]

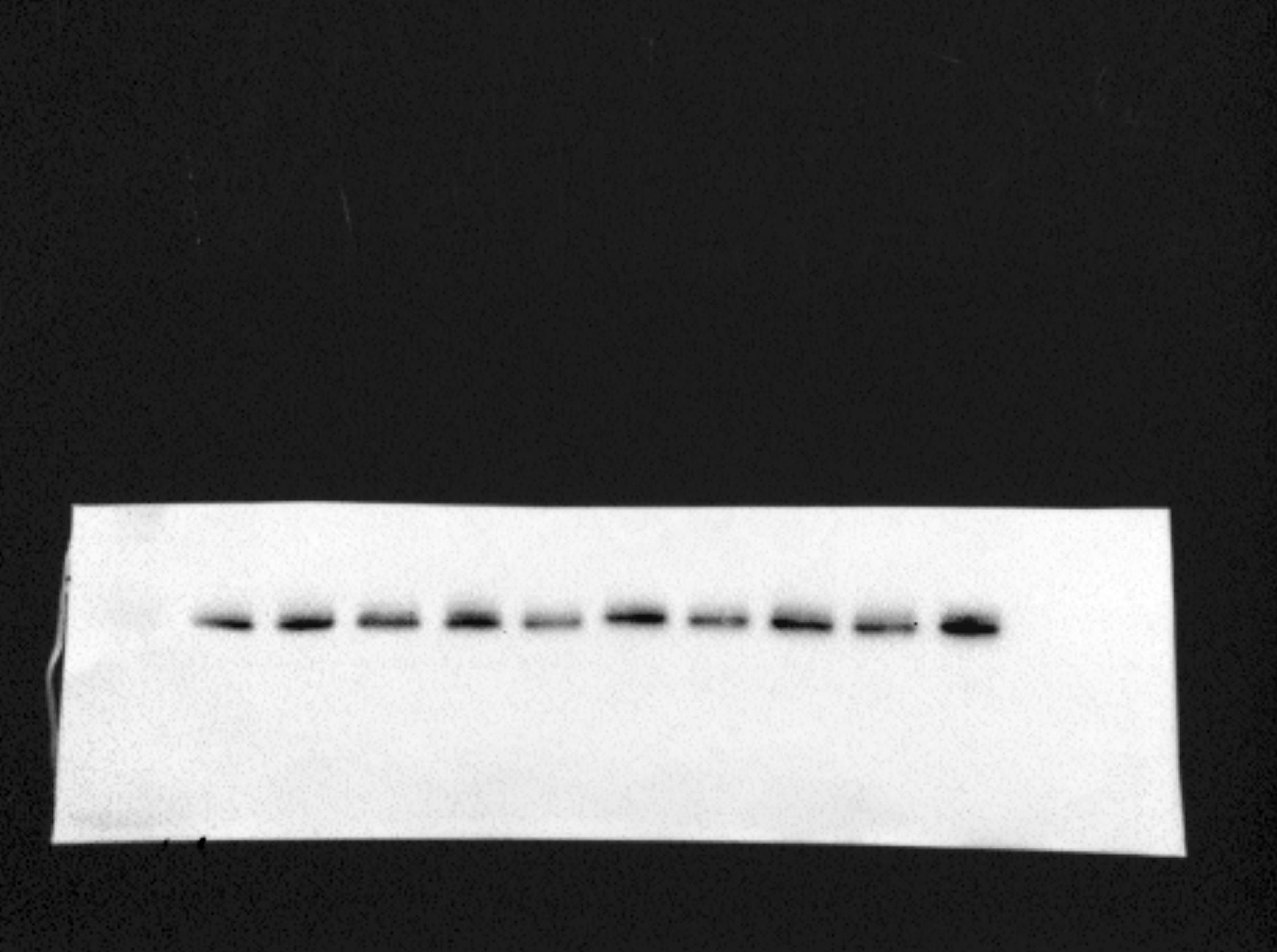

Supplement: Supplementary file 3 [file LSA-2022-01409_SdataF1.3.tif]
